# Exploring gut microbiome dysbiosis in vasculitis in adults: a systematic review

**DOI:** 10.1186/s13643-026-03359-8

**Published:** 2026-10-17

**Authors:** Kamila Hashemzadeh, Mozhdeh Ghamari, Sarah Deutsch, Andreas Schwarting, Konstantinos Triantafyllias

**Affiliations:** 1 Department of Rheumatology, Acute Rheumatology Center, Bad Kreuznach, Germany; 2 Rheumatic Disease Research Center, Mashhad University of Medical Sciences, Mashhad, Iran; 3 Division of Rheumatology and Clinical Immunology, Department of Internal Medicine I, University Medical Center of the Johannes Gutenberg University Mainz, Langenbeckstr. 1, Mainz, 55131 Germany

**Keywords:** Gut microbiota, Immune inflammation, Short-chain fatty acids, Vasculitis

## Abstract

**Background and aim:**

Although studies have confirmed that disruptions in the gut microbiota (dysbiosis) contribute to the development of autoimmune disorders, the precise pathways through which the gut microbiota influences the onset and progression of vasculitis remain largely unknown. In recent years, research has increasingly concentrated on assessing the contribution of the gut microbiota to vasculitis. This systematic study sought to assess the dominant gut microbiota profiles in vasculitis patients and compare them to those of healthy individuals.

**Methods:**

The Cochrane methodology was used to design and conduct the present systematic review. Relevant articles published up to May 2025 were searched and collected from four electronic databases: PubMed, Scopus, Medline, and Web of Science. The keywords used in the search included “vasculitis,” “Takayasu arteritis,” “giant cell arteritis,” “polyarteritis nodosa,” “microscopic polyangiitis,” “granulomatosis with polyangiitis,” “eosinophilic granulomatosis with polyangiitis,” “cryoglobulinemic vasculitis,” “IgA vasculitis,” “variable vessel vasculitis,” “Behçet’s disease,” and “Cogan’s syndrome” alongside phrases like “gut microbiome,” “gut microbiota,” and “gut flora.”

**Results:**

Overall, 12 publications were included in the analysis. Three addressed individuals with Takayasu arteritis (TAK), three investigated ANCA-associated vasculitis (AAV), another three examined Behçet’s disease (BD), while one study each pertained to IgA vasculitis (IgAV), Kawasaki disease (KD), and a nonspecific form of vasculitis. The majority of these works observed variations in both α-diversity and β-diversity, as well as in the diversity and richness of the gut microbiota. Additionally, the structure of the microbial communities differed markedly between those with vasculitis and healthy individuals. Notably, Bacteroidetes, Firmicutes, and Proteobacteria—the dominant bacterial phyla in the human gastrointestinal tract—were frequently diminished in affected patients.

**Conclusion:**

The occurrence of intestinal microbial imbalance in individuals diagnosed with vasculitis has been validated. As observed in various other autoimmune conditions, the gut microbiota of vasculitis patients is chiefly comprised Bacteroidetes, Firmicutes, Proteobacteria, and Actinobacteria. Disruptions in the microbial ecosystem—mainly typified by diminished diversity and population density—may play a role in triggering and advancing the disease.

**Systematic review registration:**

This review was not registered.

**Supplementary Information:**

The online version contains supplementary material available at https://doi.org/10.1186/s13643-026-03359-8.

## Introduction

The microbiomes naturally exist within or on the human body and have formed a symbiotic relationship with humans over time. The intestinal microbiome comprises microorganisms that inhabit the digestive tract [1]. Imbalances in gut microbiota, known as dysbiosis, can contribute to various conditions, including autoimmune disorders, by disrupting mucosal immune equilibrium and compromising gut barrier function [2, 3]. Studies have established a connection between microbiome alterations and autoimmune diseases, such as systemic lupus erythematosus and rheumatoid arthritis, highlighting its emergence as a potential therapeutic focus [4].

Epidemiological studies have shown marked geographic and ethnic variation in prevalence. For example, giant cell arteritis is the most frequent large vessel vasculitis in adults over 50 years, with an incidence of approximately 15–25 cases per 100,000 persons annually in Northern Europe and North America, but is rare in Asian populations. Takayasu arteritis, conversely, is more prevalent in Asia, particularly Japan, with an incidence of 1–2 per million annually. ANCA-associated vasculitides, including granulomatosis with polyangiitis and microscopic polyangiitis, occur at rates of 10–20 per million annually, with higher frequencies reported in Western countries for GPA and in East Asia for MPA. These variations highlight the importance of considering regional epidemiology when interpreting the burden of vasculitis [5].

Typically, the predominant enterotypes are characterized by Firmicutes, Bacteroidetes, Actinobacteria, and Proteobacteria, collectively comprising over 90% of the total gut microbiota [6]. Commensal microorganisms contribute to sustaining equilibrium and immune regulation through an ongoing and dynamic interaction with the host’s immune system [7].

A well-established mechanism facilitating this interaction involves the biochemical signaling mediated by metabolites produced by the microbiota [8]. Specifically, short-chain fatty acids (SCFAs), including butyrate, propionate, and acetate, function as inhibitors of histone deacetylase (HDAC) and as ligands for G-protein-coupled receptors (GPCRs), playing a crucial role in immune modulation [9]. Furthermore, butyrate [10] and IgA secreted by lamina propria plasma cells serve as additional elements that modulate the preservation of gut homeostasis [11].

Variations in the gut microbiota's composition, whether in diversity or abundance—referred to as dysbiosis—can disrupt the interaction between the microbiota and the immune system, leading to inflammatory responses and possibly playing a role in the development of chronic inflammatory conditions [12]. Dysregulation of gut microbiota, which plays a pivotal role in modulating immune cells like Th1, Th17, and Treg, has been linked to various autoimmune conditions and may similarly contribute to the development of vasculitis [13].

Although studies have confirmed that disruptions in the gut microbiota (dysbiosis) contribute to the development of autoimmune disorders like rheumatoid arthritis and systemic lupus erythematosus [14], the precise pathways through which the gut microbiota influences the onset and progression of IgAV remain largely unknown [15]. In recent years, research has increasingly concentrated on assessing the contribution of the gut microbiota to vasculitis. Vasculitis comprises a diverse set of autoimmune inflammatory disorders distinguished by vascular wall inflammation, perivascular tissue irritation, and necrotic cellular damage [14]. Several studies have indicated that alterations in gut microbiota significantly contribute to the development of vasculitis [10, 16–18]. The environmental factors—particularly shifts in gut microbial populations—contribute significantly to the onset of vasculitis. This imbalance in the intestinal microbiome is characterized by a heightened presence of harmful bacteria and a diminished prevalence of beneficial microbes [19]. In light of the aforementioned, this systematic study sought to assess the dominant gut microbiota profiles in vasculitis patients and compare them to those of healthy individuals.

## Materials and methods

This systematic review was conducted using methods adapted from Cochrane guidelines and focused on research investigating the gut microbiota in patients with various forms of vasculitis. The seven stages implemented in this study, consistent with the Cochrane approach, were as follows: defining the research question, establishing eligibility criteria, executing a comprehensive search, discarding studies that did not meet the criteria, evaluating study quality, extracting pertinent data, and synthesizing the findings [20]. As the majority of included studies were observational in design, the methodology was adjusted accordingly to ensure consistency and accuracy in reporting.

### Inclusion and exclusion criteria

The process of selecting articles for the review was directed by the PICO framework (Participants, Intervention, Comparison, Outcome) [21]. Eligible studies were those providing a clear methodological description, involving human participants, focusing on the gut microbiota in vasculitis, and published in English. Studies addressing autoimmune disorders other than vasculitis, those with insufficient data, animal or in vitro investigations, and narrative reports were excluded. Review articles, qualitative studies, and books were also omitted. To ensure methodological consistency, only comparative case-control studies were incorporated, while non-research articles, noncomparative studies, case reports, and case series were excluded. Studies with fewer than 20 vasculitis patients were not considered. Pediatric vasculitis studies were excluded; in mixed-age cohorts, only adult data were extracted and analyzed.

### Literature search

The search strategy was developed in collaboration with a medical information specialist, combining standardized indexing terms (MeSH) with broader free-text keywords related to vasculitis and the gut microbiome. Comprehensive searches were conducted in PubMed, Scopus, Medline, and Web of Science from inception to May 2025. In addition, reference lists of relevant articles were manually screened to identify further eligible studies. The core search terms included “vasculitis,” “Takayasu arteritis,” “giant cell arteritis,” “polyarteritis nodosa,” “microscopic polyangiitis,” “granulomatosis with polyangiitis,” “eosinophilic granulomatosis with polyangiitis,” “cryoglobulinemic vasculitis,” “IgA vasculitis,” “variable vessel vasculitis,” “Behçet’s disease,” and “Cogan’s syndrome,” combined with terms such as “gut microbiome,” “gut microbiota,” and “gut flora.”

Full reproducible search strategies for each database (including complete Boolean operators, field tags, and MeSH/controlled vocabulary where applicable) are provided in Supplementary Material S1–S4 in accordance with PRISMA 2020 guidelines. No language restrictions were applied. No formal gray literature search was performed, which is acknowledged as a limitation of this review.

### Study design and data extraction

The article selection procedure for this review followed the PICO framework. According to the PICO criteria (Population, Intervention/Exposure, Comparator, Outcome, Study Design), the inclusion parameters were defined as follows: (P) adults aged 18 years and older; (I/E) focus on the gut microbiota; (C) a comparison group without vasculitis; (O) analysis of differences in gut microbial composition between individuals with and without vasculitis; (S) comparative study formats. Only publications presented in English were eligible for the final assessment. The selection criteria required a comprehensive description of the study methodology, a focus on distinguishing gut microbiota profiles in vasculitis and non-vasculitis populations, use of human subjects, and English-language publication. This review exclusively considered comparative research, excluding other formats such as observational studies, individual case reports, and case series.

The literature review was conducted by two qualified investigators who assessed all accessible publications based on the defined research aims. Four electronic databases—PubMed, Scopus, Medline, and Web of Science—were systematically searched through the year 2025, using the aforementioned search terms. This process spanned a duration of 1 month (from April 1 to May 1, 2025). In addition, supplementary sources such as Google Scholar were consulted to locate further pertinent studies and to enhance the thoroughness of data retrieval.

At the initial stage, the investigators reviewed the titles and abstracts of the publications, eliminating those that did not align with the established inclusion criteria. If the significance of a study could not be assessed from the title and summary alone, the full manuscript was retrieved for in-depth evaluation. During the screening process, both researchers worked together to achieve consensus on which studies to include.

Following the final article selection, the complete texts were subjected to detailed examination. For research papers that were not easily obtainable, the researchers attempted to acquire the required data by contacting the designated author through email. Throughout the data extraction phase, the investigators sustained continuous dialogue. Relevant information was gathered from the selected studies in accordance with the study aims and was methodically documented using a standardized checklist.

**Fig. 1:**
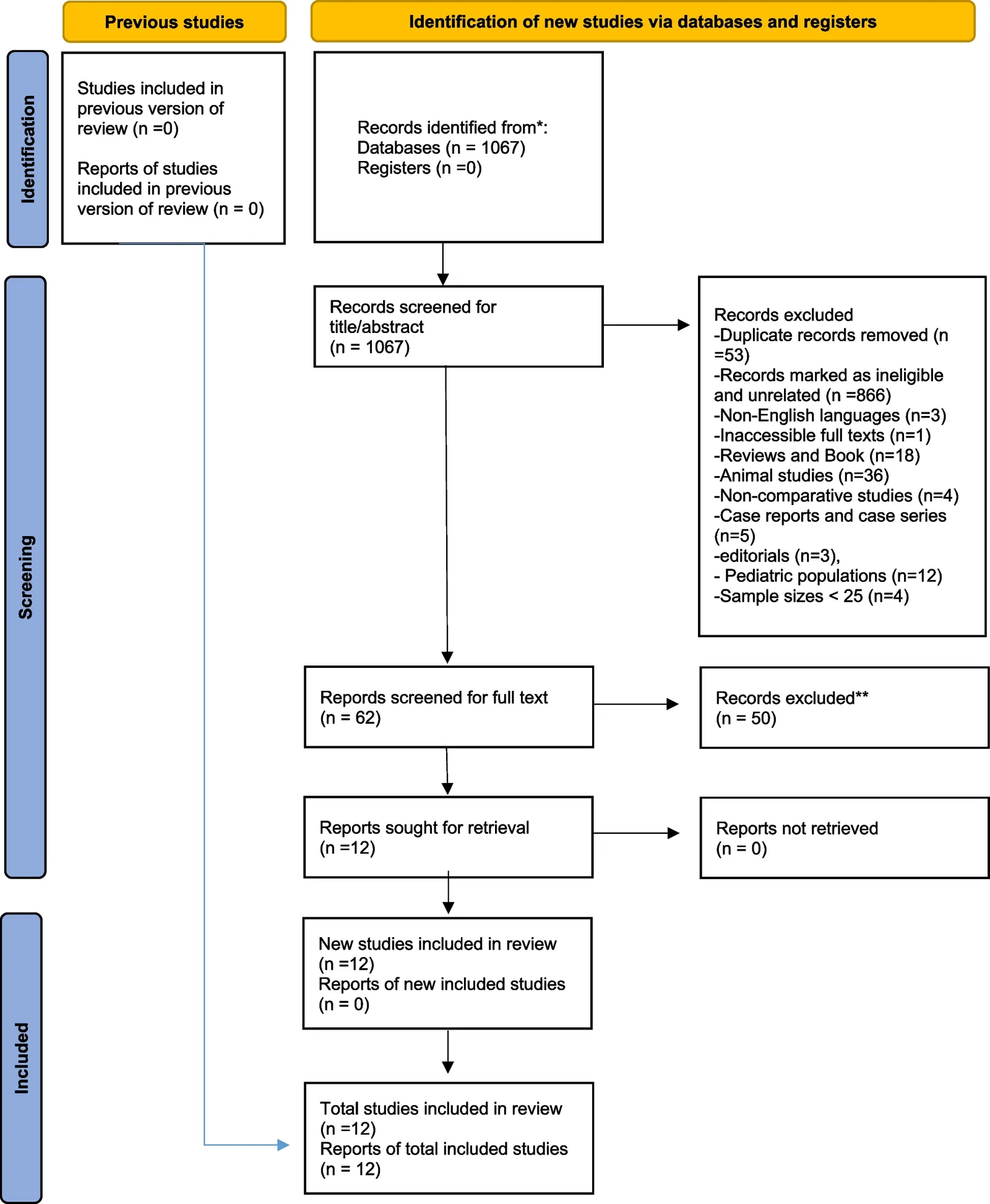
PRISMA flowchart of the article selection process

The collected data included variables such as participant count, age distribution, sex categorization, forms and classifications of vasculitis, measures of alpha and beta diversity, gut microbial richness, composition of bacterial communities, and taxonomic identification at the genus and phylum levels. Figure 1 displays a PRISMA flowchart that outlines the steps involved in selecting studies for this analysis.

### Quality assessment

The methodological quality of the included studies was assessed using the National Heart, Lung, and Blood Institute (NHLBI) Study Quality Assessment Tool for case-control studies. Each study was evaluated across key domains including clarity of the research question, definition and selection of study populations, comparability of cases and controls, exposure assessment, outcome measurement, confounding adjustment, and statistical analysis. Based on these criteria, studies were rated as good, fair, or poor quality according to the NHLBI guidance.

### Data synthesis

Due to substantial heterogeneity in study design, sample size, sequencing platforms, bioinformatic pipelines, and statistical approaches, a quantitative meta-analysis was not performed. Instead, a structured narrative synthesis was conducted to summarize and interpret the findings.

## Results

In the initial search across four databases, 1067 papers were retrieved. Of these, 866 were excluded for being irrelevant or focusing on other autoimmune conditions such as inflammatory bowel disease, rheumatoid arthritis, and systemic lupus erythematosus. After removing 53 duplicate entries, the remaining number of articles was 148.

Among these, studies published in non-English languages (*n* = 3) and those with inaccessible full texts (*n* = 1) were excluded. Additional removals included qualitative and narrative reviews (*n* = 13), animal studies (*n* = 36), and books (*n* = 5). Other exclusions involved non-comparative studies (*n* = 4), case reports and case series (*n* = 5), editorials (*n* = 3), studies conducted on pediatric populations (*n* = 12), and articles with sample sizes under 25 participants (*n* = 4).

The remaining 62 articles underwent full-text screening. At this stage, an additional 50 studies were excluded. Ultimately, 12 studies were included in the final review process (see Fig. 1).

In this review, all included studies were comparative case-control studies. In total, 515 patients with various types of vasculitis were analyzed (26 with IgA vasculitis, 26 with Kawasaki disease, 196 with Takayasu arteritis, 143 with ANCA-associated vasculitis, 91 with Behçet’s disease, and 33 with unspecified vasculitis), alongside 458 subjects in the control group. The male-to-female ratio was 1:2.18, and three studies did not report gender frequency [13, 22].

The majority of the reviewed studies were conducted in the Far East (83.3%). Specifically, 7 studies were carried out in China (58.3%), and three were performed in Japan (25%). One study was conducted in Turkey (8.3%), and one in the Netherlands (8.3%). The assessment of vasculitis classifications revealed that 5 of the 12 studies (41.7%) focused solely on small vessel vasculitis, one study (8.3%) was dedicated exclusively to medium vessel vasculitis, and three studies (25%) focused on large-vessel vasculitis.

Of the 12 studies included, one focused on IgAV, one on KD, three on TAK, two on MPA, three on BD, and two on AAV. Additionally, one study evaluated both KD and IgAV, while another addressed unspecified vasculitis. The 16S rRNA gene was utilized across all studies to characterize the bacterial lineages within the fecal microbiota. A summary of the data from these studies is provided in Table 1.

**Table 1:** Information extracted from the entered studies

Author (year) Country Reference	Sample size	Age mean or median (years)	Male/female ratio	Type of vasculitis	Vasculitis classifications	Identification tool	Alpha-diversity indexes	Beta-diversity indexes	Intestinal microbiota richness	Phylum	Genus	Bacterial community composition
Tan et al. (2022) China [23]	C: 26 H: 26	C: 25 (21.5, 40.5) H: 31 (25.8, 39.7) (*P* > 0.05)	C: 13/13 H: 13/13 (*P* > 0.05)	IgAV	Small vessel vasculitis	16S rRNA gene’s V3–V4	Lower in IgAV group compared to H group	Lower in IgAV group compared to H group	Lower in IgAV group compared to H group	Difference between two groups	IgAV patients exhibit a higher prevalence of g-*Bacteroides*, g-*Escherichia*–*Shigella*, and g-*Streptococcus*, alongside a significant decline in g-Prevotella_9	Significant difference between two groups
Teramoto et al. (2023) Japan [24]	C: 26 H: 57	C: 32.5 H: 36 (*P* > 0.05)	C: 13/13 H: 35/22 (*P* > 0.05)	KD	Medium vessel vasculitis	16S rRNA gene’s V3–V4	Significant differences only in Faith’s index	Difference between groups	–	More frequent: Firmicutes, Bacteroidota, Armatimonadota, Proteobacteria, and Verrucomicrobiota Increased: Bacteroidota Decreased: Firmicutes and Armatimonadota	More frequent: *Bacteroides*, *Faecalibacterium*, *Blautia*, and *Bifidobacterium* *Ruminococcus gnavus* was more dominant in the KD group	Significant difference between groups
Fan et al. (2023) China [25]	Discovery cohort (TAK: 57, H: 40) Validation cohort (TAK: 32, H: 24)	Discovery cohort (TAK: 38, H: 39) Validation cohort (TAK: 37.5, H: 37.4) (*P* > 0.05)	Discovery cohort (TAK: 13/44, H: 10/30) Validation cohort (TAK: 1/31, H: 1/23) (*P* < 0.05)	TAK	Large vessel vasculitis	16S rRNA genes	Significant difference between groups	Significant difference between groups	Significant difference between groups	Increases: Proteobacteria Decreased: Actinobacteria	Unclassified *Escherichia*, *Escherichia coli*, *Veillonella parvula*, *Streptococcus parasanguinis*, *Dorea*, *Bifidobacterium adolescentis*, Lachnospiraceae bacterium, *Streptococcus salivarius*, *Bifidobacterium longum*, *Bacterium*	–
Shirai et al. (2023) Japan [26]	C: 31 H: 31	C: 41 H: 41	C: 4/27 H: 4/27	TAK	Large vessel vasculitis	16S rRNA genes	Lower in TAK group	Lower in TAK group	Lower in TAK group	Microbiota changes in phylum were related with TAK	*Streptococcus*, *Lactobacillus*, *Veillonella*, *Enterococcus*, *Dialister*, *Actinomyces*, *Scardovia*, *Sellimonas*, *Rothia*, and *Campylobacter* Decreased in TAK: *Bacteroides*, *Phascolarctobacterium*, *Dorea*, *Parasutterella*, *Barnesiella*, and *Acidaminococcus*	Significant difference between two groups
Manabe et al. (2023) Japan [27]	C: 76 H: 56	C: 51 (31–68) H: 48 (34–63) (*P* > 0.05)	C: 9/67 H: 8/48 (*P* > 0.05)	TAK	Large vessel vasculitis	16S rRNA gene’s V1–V2	No difference between groups	Difference between group	–	Similar between the TAK and HC groups Increased: Streptococcaceae Decreased: Bifidobacteriaceae	Increased: *Streptococcus*, *Lactobacillus*, *Haemophilus*, *Campylobacter*, *Gemella*, and *Actinomyces* Decreased: Lachnospiraceae and *Bifidobacterium*	–
Bilge et al. (2020) Turkey [28]	C: 27 H: 10	C: 40.8 ± 9.3 H: 38.9 ± 4.9	C: 10/17 H: 4/6	BD	Small, medium, and large vasculitis	16S rRNA gene’s V3–V4	No significant difference	No significant difference	No significant difference	Actinobacteria: *Bifidobacterium*, Lactobacillaceae	Increased: *Actinomyces*, *Libanicoccus*, *Collinsella*, *Eggerthella*, *Enetrohabdus*, *Catenibacterium*, and *Enterobacter* Decreased: *Bacteroides*, *Lachnospira*, *Akkermansia*, and *Acetanaerobacterium*	Significant difference between two groups
Ye et al. (2018) China [22]	C: 32 H: 74	(*P* > 0.05)	(*P* > 0.05)	BD	Small, medium, and large vasculitis	16S rRNA gene’s V3–V4	–	Difference between groups	–	More frequent: Bacteroidetes, Firmicutes, Proteobacteria, and Actinobacteria	Increased: *Bilophila*, *Alistipes*, *Paraprevotella*, and species *Faecalibacterium prausnitzii* Decreased: *Clostridium*	Significant difference between two groups
Van der Houwen et al. (2020) Netherlands [29]	C: 32 H: 32	C: 47.96 H: 44.5	C: 15/17 H: 16/16	BD	Small, medium, and large vasculitis	16S rRNA gene’s V3–V4	No difference between groups	No difference between groups	–	*Bifidobacterium*, Lactobacillaceae Decreased: Barnesiellaceae	Decreased: unclassified Barnesiellaceae and *Lachnospira*	–
Yu et al. (2022) China [18]	C: 23 LN: 15 H: 27	C: 62 LN: 41 H: 51	C: 8/15 LN: 4/11 H: 13/14	AAV	Small vessel vasculitis	16S rRNA gene’s V3–V4	Lower in AAV compared to H group	Lower in AAV compared to H and LN groups	Lower in AAV compared to H group	More frequent in all groups: Firmicutes, Bacteroidetes, and Proteobacteria Decreased: Firmicutes and Bacteroidetes Increased: Proteobacteria	Increased: *Enterococcus*, *Peptoclostridium*, *Streptococcus*, and *Akkermansia* Decreased: *Faecalibacterium*, Prevotella_9, and *Roseburia*	Significant difference between two groups
Lee et al. (2019) China [10]	AC: 29 RC: 20 H: 14	–	–	AAV (GPA, MPA, and EGPA)	Small vessel vasculitis	16S rRNA gene’s V1–V2	–	–	–	More frequent: Firmicutes, Bacteroidetes	*Dialister*, *Prevotella*, *Faecalibacterium*, and *Sutterella* were significantly lower in active AAV compared to remission AAV	A significant difference was observed between the groups, while no variation was found between the controls and AAV remission patients
Yu et al. (2021) China [17]	AMPA: 35 IMPA: 36 H: 34	AMPA: 58 IMPA: 61 H: 61 (53–68) (*P* > 0.05)	AMPA: 12/23 IMPA: 20/16 H: 18/16 (*P* > 0.05)	MPA	Small vessel vasculitis	16S rRNA gene’s V3–V4	Low difference between groups	Difference between groups	No difference between groups	More frequent: Firmicutes, Bacteroidetes, Proteobacteria Increased: Actinobacteria and Epsilonbacteraeota	More frequent: *Bacteroides*, *Faecalibacterium*, *Blautia*, *Streptococcus*, *Escherichia*, *Shigella* Increased: *Actinomyces* and *Streptococcus* Decreased: *Subdoligranulum*, Ruminococcaceae, *Eubacterium*, *Dorea*, and *Butyricicoccus*	Significant difference between two groups
Wang et al. (2022) China [13]	C: 33 H: 33	(*P* > 0.05)	(*P* > 0.05)	Vasculitis	Small vessel vasculitis	16S rRNA genes	Lower in vasculitis group	Lower in vasculitis group	Lower in vasculitis group compared to H group	Decreased: Firmicutes Increased: Proteobacteria and Bacteroidota	Decreased: *Gemella*, *Anaeroglobus*, *Campylobacter*, *Fournierella*	Significant difference between two groups

*C* case group, *H* healthy control group, *IgAV* immunoglobulin A vasculitis, *HSP* Henoch–Schönlein purpura, *AAV* antineutrophil cytoplasmic antibody (ANCA)-associated vasculitis, *OTUs* operational taxonomic units, *KD* Kawasaki disease, *GPA* granulomatosis with polyangiitis, *MPA* microscopic polyangiitis, *EGPA* eosinophilic granulomatosis with polyangiitis, *AC* cases with active disease, *RC* remission cases, *TAK* Takayasu arteritis, *BD* Behçet’s disease, *AMPA* active microscopic polyangiitis, *IMPA* inactive microscopic polyangiitis, *IBD* intestinal Behçet’s disease, *LN* lupus nephritis

### Gut microbiome in immunoglobulin A vasculitis (Henoch–Schönlein purpura)

A single investigation centered on adult individuals diagnosed with gAV, revealing a marked decline in both the richness and heterogeneity of their intestinal microbial communities. The gender distribution among these patients was evenly split between males and females. According to findings by Tan et al., the composition of bacterial populations in IgAV cases differed significantly from that of healthy controls. Moreover, individuals with IgAV exhibited reduced levels of both α-diversity and β-diversity, in contrast to the broader and more balanced microbial profiles observed in unaffected participants. Dominant bacterial phyla identified in the gut microbiota of IgAV patients included Bacteroidetes, Firmicutes, Proteobacteria, Actinobacteria, and Fusobacteria [23].

### Gut microbiome in Takayasu arteritis

Three studies investigated the gut microbiota in patients with Takayasu arteritis (TAK), reporting a male-to-female ratio of 1:6.25. Findings consistently showed that β-diversity was lower in the TAK group compared to healthy controls [25–27]. Two of the three studies also reported a reduction in α-diversity and intestinal microbiota richness in TAK patients [25, 26], while Fan et al. [25] found no significant difference in α-diversity between the groups. Furthermore, one study did not observe a difference in bacterial community composition between TAK patients and healthy controls [26].

The most abundant gut microbiota in TAK patients included *Bifidobacterium*, *Veillonella*, *Streptococcus*, *Dorea*, Lachnospiraceae, *Lactobacillus*, *Actinomyces*, and *Campylobacter* [25–27]. At the phylum level, Fan et al. [25] reported an increase in Proteobacteria and a decrease in Actinobacteria in TAK patients compared to healthy controls. Manabe et al. [27] observed an increase in Streptococcaceae and a decrease in Bifidobacteriaceae in TAK patients. Additionally, Shirai et al. [26] and Manabe et al. [27] found elevated levels of *Streptococcus*, *Lactobacillus*, and *Actinomyces*. A decrease in Lachnospiraceae and *Bifidobacterium* levels was reported in two studies [25, 27].

### Gut microbiome in Behçet’s disease

Three of the included studies explored the gut microbiota in individuals with BD, with a male-to-female ratio of 1:1.36 among participants. Two of the three analyzed studies did not identify a significant difference between the BD and control groups in terms of α-diversity, β-diversity, and intestinal microbiota richness [28, 29]. However, Ye et al. [22] reported a notable variation in β-diversity between BD patients and healthy individuals.

Both Bilge et al. and Ye et al. [22, 28] found substantial differences in bacterial community composition between BD patients and healthy controls. Two investigations highlighted an increased presence of Actinobacteria, specifically *Bifidobacterium* and Lactobacillaceae, in BD patients [28, 29]. Additionally, Ye et al. [22] observed a higher abundance of Bacteroidetes, Firmicutes, Proteobacteria, and Actinobacteria in BD patients. Regarding specific bacterial changes, Van der Houwen et al. [29] and Bilge et al. [28] documented a decline in *Lachnospira* among BD patients, while Ye et al. [22] noted a reduction in *Clostridium* levels in this group.

### Gut microbiome in ANCA-related vasculitis

A total of three studies assessed the intestinal microbiota in individuals diagnosed with ANCA-associated vasculitis (AAV), reporting a male-to-female ratio of 1:1.35 among participants. Two of the three studies identified a significant disparity between AAV patients and healthy controls regarding α-diversity and β-diversity [17, 18]. However, Yu et al. [17] reported no notable variation in intestinal microbiota richness between the two groups.

All three studies observed a considerable difference in bacterial community composition between AAV patients and healthy individuals. Although Lee et al. [10] detected a distinct contrast in bacterial composition between AAV patients and controls, no significant divergence was noted between healthy controls and AAV patients in remission.

Firmicutes, Bacteroidetes, and Proteobacteria were the predominant gut microbiota in AAV patients [10, 17, 18]. Two studies revealed a reduction in *Faecalibacterium*, Prevotella_9, *Roseburia*, *Subdoligranulum*, Ruminococcaceae, *Eubacterium*, *Dorea*, and *Butyricicoccus*, while *Streptococcus*, *Enterococcus*, *Actinomyces*, and *Akkermansia* exhibited an increase in AAV patients [17, 18]. Additionally, Lee et al. [10] documented a significant decrease in *Dialister*, *Prevotella*, *Faecalibacterium*, and *Sutterella* in active AAV compared to those in remission.

### Gut microbiome in Kawasaki disease

A single study investigated the gut microbial profile in individuals diagnosed with Kawasaki disease (KD), with an equal distribution of male and female participants. Findings from Van der Houwen et al. revealed diminished alpha-diversity within the KD group relative to healthy controls. Likewise, beta-diversity was notably reduced, and overall microbial richness was significantly lower among KD patients. The composition of gut bacteria in KD cases also displayed marked differences compared to the control group. Moreover, the study identified an elevated presence of Bacteroidota, alongside reduced levels of Firmicutes and Armatimonadota in the intestinal microbiota of those with KD [29].

#### Quality assessment

Overall, the included studies were predominantly observational case–control or cohort designs with moderate methodological quality. Most studies appropriately matched patients and healthy controls for key demographic variables such as age and sex, and excluded major confounders including recent antibiotic use and comorbid inflammatory or metabolic diseases. Microbiome profiling was mainly performed using 16S rRNA gene sequencing, with a subset of studies applying shotgun metagenomics and functional prediction tools such as PICRUSt2 or KEGG-based annotation. Despite these strengths, several limitations were consistently observed across studies. Sample sizes were generally small, and there was considerable heterogeneity in study populations, disease activity status, and geographic origin. In addition, variability in sequencing platforms, bioinformatic pipelines, and taxonomic classification methods introduced methodological inconsistency. Most studies were cross-sectional in design, limiting causal inference. Furthermore, adjustment for dietary factors, medication use, and other environmental confounders was incomplete in several datasets. Overall, while the studies provide consistent evidence of disease-associated alterations in the gut microbiome, the certainty of evidence is limited by risk of bias, small sample sizes, and methodological heterogeneity, resulting in an overall moderate quality of evidence. The findings of the quality evaluation are illustrated in Table 2.

**Table 2:** Quality assessment of the studies included in this review

Study	Clearly stated question	Study population	Selection bias	Sample size justification	Exposure measured prior to outcome	Valid exposure measurement	Valid outcome measurement	Blinding	Confounding adjustment	Missing data	Confounder measurement	Selective reporting	Other bias	Overall quality
Tan et al. [23]	Yes	Yes	Unclear	Yes	Yes	Yes	Yes	Unclear	No	Unclear	Partial	Unclear	No	Fair
Li et al. [30]	Yes	Yes	Unclear	No	Yes	Yes	Yes	Unclear	No	Yes	Yes	Unclear	Yes	Fair
Yu et al. [18]	Yes	Yes	Unclear	No	Yes	Yes	Yes	Unclear	No	Yes	Yes	Unclear	Yes	Fair
Bilge et al. [28]	Yes	Yes	Unclear	No	Yes	Yes	Yes	Unclear	No	Yes	Yes	Unclear	Yes	Fair
Wang et al. [13]	Yes	Yes	Unclear	No	Yes	Yes	Yes	Unclear	No	Yes	Yes	Unclear	Yes	Fair
Fan et al. [25]	Yes	Yes	Unclear	Yes	Yes	Yes	Yes	Unclear	Yes	Yes	Yes	Unclear	No	Fair
Shirai et al. [26]	Yes	Yes	Unclear	No	Yes	Yes	Yes	Unclear	No	Yes	Unclear	Yes	No	Fair
Manabe et al. [27]	Yes	Yes	Unclear	No	Yes	Yes	Yes	Unclear	Yes	Yes	Yes	Unclear	Moderate risk	Moderate
Yu et al. [17]	Yes	Yes	Unclear	No	Yes	Yes	Yes	No	Partial	Unclear	Yes	Unclear	Yes (medication + renal severity confounding)	Fair
Ye et al. [22]	Yes	Yes	Unclear	No	Yes	Yes	Yes	No	Partial	Unclear	Yes	No	Yes (FMT + fungal overrepresentation + heterogeneity BD activity)	Good
Teramoto et al. [24]	Yes	Yes	Unclear	Yes	Yes	Yes	Yes	No	No	Unclear	Partial	Low–unclear	Moderate (age, delivery mode, diet-related bias)	Fair
Van der Houwen et al. [29]	Yes	Yes	Unclear	No	Yes	Yes	Yes	No	No	Unclear	Partial	Low–unclear	Moderate (country effect, meds, IgA subanalysis bias)	Fair

## Discussion

### Gut microbiome in immunoglobulin A vasculitis (Henoch–Schönlein purpura)

Immunoglobulin A vasculitis (IgAV) is uncommon in adults, and only one study investigating gut microbiota in adult patients was identified. Tan et al. reported reduced α-diversity and significant differences in β-diversity in IgAV patients compared with healthy controls, indicating altered microbial richness and composition [23]. Tan et al. also identified reductions in *Prevotella*, *Roseburia*, and *Streptococcus* in IgAV patients [23]. *Prevotella* has been associated with anti-inflammatory and nephroprotective effects, while *Roseburia* is an important butyrate-producing genus [23, 31]. Although *Streptococcus* has been linked to IgAV exacerbation in previous studies, its abundance was reduced in the gut microbiota of IgAV patients in the study by Tan et al. [23, 32]. These findings suggest that alterations in specific microbial taxa may contribute to immune dysregulation in IgAV. Patients with recurrent IgAV showed a significantly higher abundance of *Escherichia*–*Shigella* than healthy controls, and its enrichment was associated with disease recurrence [23]. Dysbiosis and host inflammatory responses may promote the expansion of Enterobacteriaceae, potentially contributing to disease progression [33].

### Gut microbiome in ANCA-associated vasculitis

ANCA-associated vasculitis (AAV), including microscopic polyangiitis (MPA), granulomatosis with polyangiitis (GPA), and eosinophilic granulomatosis with polyangiitis (EGPA), has been associated with alterations in gut microbiota. Yu et al. reported reduced microbial abundance and diversity in AAV patients with renal impairment compared with healthy individuals, along with increased *Akkermansia*, Ruminococcaceae, *Lactobacillus*, and *Enterococcus* and decreased Lachnospiraceae_NK4A136_group [18]. Gut dysbiosis may also contribute to renal injury through increased production of uremic toxins, renal fibrosis, and oxidative stress [34].

Microbial alterations have also been associated with disease activity. Yu et al. and Lee et al. reported changes in *Dialister*, *Prevotella*, *Faecalibacterium*, and *Sutterella* between active and remission phases of AAV, with *Dialister* abundance associated with higher BVAS/WG scores [10, 17]. Chen et al. observed reductions in butyrate-producing bacteria, including *Roseburia*, *Eubacterium hallii*, *Anaerostipes*, and *Butyricicoccus*, which may be relevant given the anti-inflammatory role of butyrate [10, 35–37]. Yu et al. additionally reported increased *Eggerthella* and decreased *Lachnospira*, while Fusobacteria, Actinobacteria, and Epsilonbacteraeota were more abundant in MPA than in active MPA and healthy controls [17]. *Actinomyces* and *Streptococcus* were consistently enriched across several studies [10, 38, 39].

### Gut microbiome in Takayasu arteritis

Microbial imbalance in TAK is linked to increased inflammation and complications. Shirai et al. reported significant disruption of gut microbiota in TAK, with reduced fecal diversity and depletion of genera such as *Bacteroides*, *Phascolarctobacterium*, *Dorea*, *Parasutterella*, *Barnesiella*, and *Acidaminococcus* [26]. Manabe et al. found gut microbiota alterations in TAK that may predict aneurysm expansion. This imbalance was marked by increased oral-origin bacteria (*Streptococcus*, *Campylobacter*) and was present even in untreated patients [27]. Numerous investigations exploring the connection between microbes and large-artery vasculitis (LVV) have been conducted [40–42]. In TAK cases, an elevated presence of *Bdellovibrio* and Cytophagaceae was noted, whereas a diminished prevalence of *Staphylococcus* and *Hyphomicrobium* was observed in comparison to healthy individuals [42].

Regarding *Campylobacter*, Manabe et al. [27] reported higher abundance, while Wishart [8] found no difference compared to healthy controls. Differences in patient populations (treated vs. newly diagnosed) may explain this discrepancy [25]. Species-level analysis identified *C. gracilis* (formerly *Bacteroides gracilis*) as the driver [36], with links to brain aneurysms [37]. Proton pump inhibitor use was associated with increased *Streptococcus* and *Campylobacter* in the gut [43–45].

Fan et al. also reported enrichment of *Escherichia* and *Klebsiella pneumoniae* in TAK, both implicated in immune-mediated inflammation [46, 47]. L. Bacterium 7 1 58FAA, previously isolated from inflamed colon tissue and linked to IBS pain, was enriched in active TAK [29, 43].

### Gut microbiome in Behçet’s disease

Patients with BD consistently show distinct microbial profiles compared to healthy individuals, suggesting that dysbiosis may play a role in disease activity [22, 28, 29]. For example, Bilge et al. demonstrated that BD patients harbor higher levels of *Actinomyces*, *Collinsella*, and *Eggerthella*, while beneficial genera such as *Lachnospira*, Ruminococcaceae, and *Acetanaerobacterium* are reduced [28]. These shifts are not uniform across all patients, as different clinical forms of BD present unique microbial signatures, a finding supported by other investigations [48–50]. Among these taxa, *Collinsella* stands out as particularly abundant in BD, and its presence has been linked to HLA variants associated with inflammatory disease [51]. Interestingly, *Collinsella* abundance correlates with insulin levels and is negatively influenced by dietary fiber intake, suggesting that nutritional habits can directly shape microbial composition and, in turn, metabolic and inflammatory health [52].

Complementing these observations, Ye et al. reported that opportunistic pathogens and sulfate-reducing bacteria (SRB) are enriched in BD, whereas butyrate-producing bacteria (BPB) and methanogens are more common in healthy individuals [22, 49, 53]. Since BPB and methanogens help sustain immune balance, their depletion—driven by the expansion of SRB, *Stenotrophomonas*, *Actinomyces*, and *Paraprevotella*—may exacerbate inflammation, particularly in active BD [54, 55].

Other studies highlight reductions in protective taxa. Van et al. found diminished Barnesiellaceae and *Lachnospira* in BD patients, a pattern associated with heightened TNF-α levels and colitis in experimental models [56, 57]. This suggests that loss of these microbes may remove a natural brake on inflammatory cytokines central to BD. Similarly, Cabn et al. identified IgA-coated *Bifidobacterium*, *Ruminococcus bromii*, and *Dorea* in BD, pointing to an immune system that recognizes and responds to these microbes as potential pathosymbionts [53, 58].

Taken together, these findings suggest that BD is not driven by a single microbial or metabolic abnormality. Instead, the disease reflects a complex interplay between genetic susceptibility, dietary influences, and shifts in microbial composition. Dysbiosis in BD appears to weaken protective microbial functions while amplifying pro-inflammatory taxa, thereby shaping immune cell differentiation and contributing to the vascular and gastrointestinal manifestations of the disease.

### Gut microbiome in Kawasaki disease

Recent investigations have reported modifications in the structure of the gut microbiota among subjects diagnosed with KD [59, 60]. Several datasets suggest that the relative abundance of *Ruminococcus*, *Blautia*, *Faecalibacterium*, and *Roseburia* significantly increased during the subacute phase of the disease [61, 62]. Similarly, Teramoto and colleagues presented findings indicating a higher representation of the *Ruminococcus gnavus* cluster within the intestinal microbial community. Moreover, a diminished concentration of *Blautia* in the gut flora may function as a potential biomarker in individuals with a history of KD [24]. The *Ruminococcus gnavus* group produces a complex carbohydrate linked to the stimulation of inflammatory pathways [63]. Several lines of evidence have reported a decline in beneficial microbial groups, such as *Ruminococcus* and *Faecalibacterium*, in individuals diagnosed with KD [64]. In children with a prior history of KD, the development of beneficial gut bacterial communities may be impeded. As a result, an atypical mucosal immune reaction may arise. Therefore, dysbiosis could represent a contributing factor to the pathogenesis of KD [24].

## Limitations

This comprehensive analysis provides important perspectives on the gut microbiome in individuals with vasculitis. However, it is noteworthy that the review did not incorporate any randomized controlled trials. This analysis included only case–control studies with adequate sample sizes, while those with limited participant numbers were excluded. As a result, the findings from these investigations can be considered largely reliable. A major limitation of this research, however, is the scarcity of studies with large sample sizes, partly due to the rare occurrence of vasculitis. Moreover, because most of the included studies were conducted at single centers, the generalizability of the results to broader populations is restricted. Although all investigations in this review examined the gut microbiome in individuals with vasculitis, evaluating the effects of microbiome-related treatments was difficult due to inconsistencies among the studies. In addition, variations in vasculitis subtypes introduced substantial heterogeneity, making a meta-analysis impractical.

## Conclusion

Most studies investigating intestinal flora in vasculitis have consistently reported the presence of gut microbiota dysbiosis in affected patients. Similar to other autoimmune disorders, the intestinal microbiota of patients with vasculitis is predominantly composed of Bacteroidetes, Firmicutes, Proteobacteria, and Actinobacteria. Observed imbalances in gut microbiota, primarily characterized by reduced diversity and abundance, are associated with vasculitis and may be relevant to its clinical course. While these findings highlight a potential link between dysbiosis and vasculitis, the observational nature of the available studies precludes causal inference.

## Supplementary Information

Additional file 1: PRISMA 2020 Checklist. This file includes the officially completed PRISMA Checklist, available at https://www.prisma-statement.org/prisma-2020-checklist.

Additional file 2: PRISMA 2020 for Abstracts Checklist. This file includes the officially completed PRISMA Checklist for Abstracts, available at https://www.prisma-statement.org/abstracts.

Additional file 3: Electronic database search strategy.

## Data Availability

All data generated or analyzed during this study are included in this published article or are available from the corresponding author on reasonable request.

## Abbreviations

TAK: Takayasu arteritis
AAV: ANCA-associated vasculitis
BD: Behçet’s disease
IgAV: Immunoglobulin A vasculitis
KD: Kawasaki disease
SCFAs: Short-chain fatty acids
HDAC: Histone deacetylase
GPCRs: G-protein-coupled receptors
MPA: Microscopic polyangiitis
GPA: Granulomatosis with polyangiitis
EGPA: Eosinophilic granulomatosis with polyangiitis
BVAS/WG: Wegener’s granulomatosis
SRB: Sulfate-reducing bacteria
BPB: Butyrate-producing bacteria
CD: Crohn’s disease
Treg: Regulatory T cells
