# Supplementary material for: Exploring gut microbiome dysbiosis in vasculitis in adults: a systematic review
Source: Syst Rev. 2026 Oct 17;15:272. doi: 10.1186/s13643-026-03359-8 (PMC13676004; doi:10.1186/s13643-026-03359-8)
Supplement: Supplementary file 3 [file 13643_2026_3359_MOESM3_ESM.docx]

Electronic Database Search Strategy

**Exploring Gut Microbiome Dysbiosis in Vasculitis in adults: A Systematic Review**

This document presents reproducible search strategies for the four databases named in the manuscript. The searches covered database inception through May 2025.

| **Database** | **Platform / Search Field** | **Records Before Deduplication** |
| --- | --- | --- |
| PubMed | PubMed, MeSH + title/abstract | 272 |
| Scopus | Scopus, TITLE-ABS-KEY | 318 |
| MEDLINE | Ovid MEDLINE, Subject Headings + ti,ab,kf | 225 |
| Web of Science Core Collection | Web of Science, Topic (TS) | 252 |
| Total | All databases, before deduplication | 1067 |

**S1. Pubmed**

| **Database / platform** | PubMed |
| --- | --- |
| **Search coverage** | Database inception to May 2025 |
| **Displayed result count** | **272** |

Fields searched: MeSH terms and title/abstract.

Full Search Query

```
(
  "Vasculitis"[Mesh] OR vasculitis[tiab] OR "Takayasu Arteritis"[Mesh] OR "Takayasu arteritis"[tiab]
  OR "Giant Cell Arteritis"[Mesh] OR "giant cell arteritis"[tiab]
  OR "Polyarteritis Nodosa"[Mesh] OR "polyarteritis nodosa"[tiab]
  OR "Microscopic Polyangiitis"[Mesh] OR "microscopic polyangiitis"[tiab]
  OR "Granulomatosis with Polyangiitis"[Mesh] OR "granulomatosis with polyangiitis"[tiab]
  OR "Wegener granulomatosis"[tiab]
  OR "Eosinophilic Granulomatosis with Polyangiitis"[Mesh] OR "eosinophilic granulomatosis with polyangiitis"[tiab]
  OR "Churg-Strauss syndrome"[tiab]
  OR "Cryoglobulinemia"[Mesh] OR "cryoglobulinemic vasculitis"[tiab]
  OR "Immunoglobulin A Vasculitis"[Mesh] OR "IgA vasculitis"[tiab] OR "Henoch-Schonlein purpura"[tiab]
  OR "Behcet Syndrome"[Mesh] OR Behcet*[tiab] OR Behçet*[tiab]
  OR "Cogan Syndrome"[Mesh] OR "Cogan syndrome"[tiab]
  OR "Kawasaki Disease"[Mesh] OR "Kawasaki disease"[tiab]
  OR "ANCA-associated vasculitis"[tiab] OR "variable vessel vasculitis"[tiab]
)
AND
(
  "Gastrointestinal Microbiome"[Mesh] OR "Microbiota"[Mesh]
  OR "gut microbiome"[tiab] OR "gut microbiota"[tiab] OR "gut flora"[tiab]
  OR "intestinal microbiome"[tiab] OR "intestinal microbiota"[tiab] OR "intestinal flora"[tiab]
  OR "fecal microbiota"[tiab] OR "faecal microbiota"[tiab] OR dysbiosis[tiab]
)
NOT (animals[mh] NOT humans[mh])
```

**S2. Scopus**

| **Database / platform** | Scopus |
| --- | --- |
| **Search coverage** | Database inception to 1 May 2025 |
| **Displayed result count** | **318** |

Full Search Query

```
TITLE-ABS-KEY(
  vasculitis OR "Takayasu arteritis" OR "giant cell arteritis" OR "polyarteritis nodosa"
  OR "microscopic polyangiitis" OR "granulomatosis with polyangiitis" OR "Wegener granulomatosis"
  OR "eosinophilic granulomatosis with polyangiitis" OR "Churg-Strauss syndrome"
  OR "cryoglobulinemic vasculitis" OR "IgA vasculitis" OR "Henoch-Schonlein purpura"
  OR "Behcet disease" OR "Behçet disease" OR "Cogan syndrome" OR "Kawasaki disease"
  OR "ANCA-associated vasculitis" OR "variable vessel vasculitis"
)
AND TITLE-ABS-KEY(
  "gut microbiome" OR "gut microbiota" OR "gut flora" OR "intestinal microbiome"
  OR "intestinal microbiota" OR "intestinal flora" OR "fecal microbiota" OR "faecal microbiota" OR dysbiosis
)
AND NOT TITLE-ABS-KEY(animal OR mice OR mouse OR murine OR rat OR rats)
```

**S3. MEDLINE**

| **Database / platform** | Ovid MEDLINE |
| --- | --- |
| **Search coverage** | Database inception to 1 May 2025 |
| **Displayed result count** | **225** |

Fields searched: Ovid Subject Headings and title, abstract and keyword heading word (ti,ab,kf).

Full Search Query

1. exp Vasculitis/ OR vasculitis.ti,ab,kf.
2. Takayasu Arteritis/ OR "Takayasu arteritis".ti,ab,kf.
3. Giant Cell Arteritis/ OR "giant cell arteritis".ti,ab,kf.
4. Polyarteritis Nodosa/ OR "polyarteritis nodosa".ti,ab,kf.
5. Microscopic Polyangiitis/ OR "microscopic polyangiitis".ti,ab,kf.
6. Granulomatosis with Polyangiitis/ OR "granulomatosis with polyangiitis".ti,ab,kf. OR "Wegener granulomatosis".ti,ab,kf.
7. Eosinophilic Granulomatosis with Polyangiitis/ OR "eosinophilic granulomatosis with polyangiitis".ti,ab,kf. OR "Churg-Strauss syndrome".ti,ab,kf.
8. Cryoglobulinemia/ OR "cryoglobulinemic vasculitis".ti,ab,kf.
9. Immunoglobulin A Vasculitis/ OR "IgA vasculitis".ti,ab,kf. OR "Henoch-Schonlein purpura".ti,ab,kf.
10. Behcet Syndrome/ OR Behcet*.ti,ab,kf. OR Behçet*.ti,ab,kf.
11. Cogan Syndrome/ OR "Cogan syndrome".ti,ab,kf.
12. Kawasaki Disease/ OR "Kawasaki disease".ti,ab,kf.
13. "ANCA-associated vasculitis".ti,ab,kf. OR "variable vessel vasculitis".ti,ab,kf.
14. or/1-13
15. Gastrointestinal Microbiome/ OR Microbiota/
16. (gut adj3 (microbiom* OR microbiot* OR flora)).ti,ab,kf.
17. (intestinal adj3 (microbiom* OR microbiot* OR flora)).ti,ab,kf.
18. ((fecal OR faecal) adj3 microbiot*).ti,ab,kf. OR dysbiosis.ti,ab,kf.
19. or/15-18
20. 14 and 19
21. exp Animals/ not Humans/
22. 20 not 21

**S4. Web of Science Core Collection**

| **Database / platform** | Web of Science Core Collection |
| --- | --- |
| **Search coverage** | Database inception to 1 May 2025 |
| **Displayed result count** | **252** |

Field searched: Topic (TS; title, abstract, author keywords and Keywords Plus).

Full Search Query

```
TS=(
  vasculitis OR "Takayasu arteritis" OR "giant cell arteritis" OR "polyarteritis nodosa"
  OR "microscopic polyangiitis" OR "granulomatosis with polyangiitis" OR "Wegener granulomatosis"
  OR "eosinophilic granulomatosis with polyangiitis" OR "Churg-Strauss syndrome"
  OR "cryoglobulinemic vasculitis" OR "IgA vasculitis" OR "Henoch-Schonlein purpura"
  OR "Behcet disease" OR "Behçet disease" OR "Cogan syndrome" OR "Kawasaki disease"
  OR "ANCA-associated vasculitis" OR "variable vessel vasculitis"
)
AND TS=(
  "gut microbiome" OR "gut microbiota" OR "gut flora" OR "intestinal microbiome"
  OR "intestinal microbiota" OR "intestinal flora" OR "fecal microbiota" OR "faecal microbiota" OR dysbiosis
)
NOT TS=(animal OR mice OR mouse OR murine OR rat OR rats)
```
